# Heterogeneous Diffusion of Government Microblogs and Public Agenda Networks during Public Policy Communication in China

**DOI:** 10.3390/e25040640

**Published:** 2023-04-11

**Authors:** Meng Cai, Xue Gong, Jiaqi Liu

**Affiliations:** School of Humanities and Social Sciences, Xi’an Jiaotong University, Xi’an 710049, China

**Keywords:** government microblogs agenda networks, public agenda networks, heterogeneous diffusion, public policy, entropy

## Abstract

During public policy information diffusion, policy interpretation on government microblogs and public attention interact, but there are certain differences. We construct a research framework for the heterogeneous diffusion of public policy information on government microblogs. An empirical study is conducted based on the Network Agenda Setting (NAS) model. First, a combination of topic mining and content analysis is used to identify the issues discussed by government microblogs and citizens. Then, we use the importance of nodes in Degree Structure (DS) and Flow Structure (FS) entropy to measure their attention to different issues. Finally, the Quadratic Assignment Procedure (QAP) correlation and regression analysis explore the degree of heterogeneity and causal relationship between government microblog agenda networks (GMANs) and public agenda networks (PANs). We find that GMANs influence PANs and the degree of heterogeneity between them is relatively low at the beginning of policy implementation. However, as government microblogs reveal positive effects of policy implementation, they fail to influence PANs effectively, and there is a greater degree of heterogeneity between them. Moreover, PANs do not significantly affect GMANs. The dynamic leading relationship between GMANs and PANs in public policy diffusion is clarified, helping to shape the image of digital government in public opinion.

## 1. Introduction

Promoting and strengthening digital government has been a major policy objective of governments worldwide since the mid-1990s [1]. Meanwhile, social attention to digital government continues to grow in China. From 2017 to 2021, China’s internet users grew from 772 million to 1.032 billion, and the internet penetration rate rose from 55.8% to 73% [2]. In the era of intelligent media, Sina microblog, the biggest microblog platform in China, has become an essential platform for citizens’ communication and even the dissemination of public policy information. The size of China’s Internet government service users has reached 921 million, accounting for 89.2% of the total Internet users by December 2021 [3]. Therefore, microblog is being widely used by citizens and government departments.

Government new media, represented by government microblogs, provide a large capacity for information and offer a range of channels for government–citizen interactions, thereby facilitating public participation [4]. Government microblogs facilitate policy dissemination, making government work more transparent and policy information more readily available and updated. Digital government services are also greatly enhanced [5]. However, the multiple perspectives and fragmented interpretations often lead to information bias in policy information diffusion. Policy interpretation on government microblogs and public attention interact, but there are specific differences, i.e., agenda heterogeneity [6]. Isomerization as a chemical concept refers mainly to a chemical reaction in which the molecular weight remains the same while the molecular structure changes. Drawing on molecular structuring in the natural sciences to conceptualize the restructuring of issues in policy information diffusion, Xiang et al. propose the concept of agenda heterogeneity, i.e., the information bias that occurs in public policy diffusion, where the policy itself, media propaganda, and public attention influence each other but are heterogeneous [7]. However, existing studies on public policy communication mainly focus on the difference between the policy and public concern [8], and for the government social media, they mainly pay attention to the policy communication strategy and communication effect [9], ignoring the agenda-setting effect and the guiding relationship between government microblogs and the public. Therefore, we focus on the agenda heterogeneity between the GMANs and PANs.

The omnidirectional flow of information in the Internet era [10] and the changes in the understanding of the human cognitive structure, i.e., the human cognitive structure is close to the network structure and is not linear [11]. Scholars in different fields are driven to explore the relationship between media agenda and public agenda networks widely based on NAS theory [12,13]. Only a few studies focus on the agenda-setting effect and the guiding relationship between government social media and the public in crises [14,15]. There is no consistent result on the agenda-leading relationship between the two. Therefore, we explore the role of government microblogs in agenda-setting and the agenda-leading relationship between government microblogs and the public based on NAS theory in public policy communication.

In addition, at the level of research methodology, existing studies mainly use traditional centrality measures, e.g., degree centrality, to measure the importance of different issues in agenda networks [16]. This approach overemphasizes the local characteristics of the network and ignores the global topology of the network. Therefore, we draw on the idea of bridges in the study of social network information diffusion and use FS entropy to comprehensively measure the structural importance of issues in the agenda network by combining medial and radial measures. See Section 3.5.2 for more details.

Overall, this study focuses on the following research questions: In discussing specific public policies on social media, (1) What issues do government microblogs and the public focus on, and how do they differ in their attention to different issues? (2) What is the correlational relationship between GMANs and PANs, and to what extent are they heterogeneous? (3) Do GMANs effectively guide PANs or vice versa? Clarifying the above research questions can provide an essential guide for government departments to adjust the direction of policy coverage on government microblogs promptly. Therefore, we propose a research framework for the heterogeneous diffusion of public policy information, viewed through the heterogenization between GMANs and PANs. Based on government microblogs and public online discussion text data, we first use the LDA model to explore the different object agendas of the two. To mitigate the limitations of machine modeling, we combine the highly similar topics returned by the model using manual coding. We then use the social network analysis to construct their co-occurrence matrix and visualize the corresponding agenda networks. Meanwhile, we use the importance of nodes in DS and FS entropy to measure their attention to different issues. Moreover, we use QAP correlation analysis to examine the relationship between the two agenda networks during the same period and QAP regression analysis to explore how the government microblog and the public impact influence other agenda networks. Finally, we conduct an empirical study on the divorce cooling-off period policy to precisely measure the heterogeneous diffusion of public policy information, considering different subjects’ topic propagation mechanisms, the relationship, and the mutual influence between different subjects’ agenda networks. In summary, this study makes the following contributions:

- As far as we know, this is the first attempt to construct a research framework for the heterogeneous diffusion of public policy information on government microblogs. The proposed research framework can guide the public policy information diffusion on government microblogs, reduce the degree of heterogeneity between government microblogs and public attention to public policy, and contribute to policy implementation and shaping the image of democratic governments.
- This study combines issue importance measures from information entropy with the traditional degree centrality, i.e., FS and DS entropy, to comprehensively measure the differences between GMANs’ and PANs’ attention to different issues. Meanwhile, we integrate the classical agenda-setting research methods, i.e., QAP correlation and regression analysis, to comprehensively measure the heterogeneous diffusion phenomenon between government microblogs and the public from three dimensions: differences in issue attention, degree of agenda heterogeneity, and agenda causality.
- The network agenda-setting theory is extended to public policy information diffusion on government microblogs. Extending from a single temporal cross-section to a time series perspective, we analyze the agenda-leading relationship between GMANs and PANs.

The remainder of this study is organized as follows. Section 2 briefly reviews the heterogeneous diffusion of policy information, three levels of agenda-setting theory, public policy communication and NAS, and information diffusion on social networks and NAS. The research methodology and procedure are detailed in Section 3. The differences in issue attention, degree of agenda heterogeneity, and agenda causality between GMANs and PANs are presented in Section 4. In Section 5, we provide a discussion. Conclusion remarks are outlined in Section 6.

## 2. Literature Review

### 2.1. Heterogeneous Diffusion of Policy Information

Policy information diffusion refers to transferring of policy information between organizations and individuals [17]. The quality of policy information diffusion significantly affects the effectiveness of policy implementation [18]. Effective policy information diffusion can enhance public understanding, recognition, and support for policies, reduce resistance during policy implementation, and facilitate the establishment of good government–public relations. However, existing studies have indicated heterogeneity in policy information diffusion within China [19,20]. Exploring the degree and specific manifestations of heterogeneous diffusion of policy information among different subjects can help governments better adjust policy dissemination strategies, enhance citizens’ acceptance of the policy, and promote effective policy implementation.

Currently, studies on the heterogeneous diffusion of policy information are mainly conducted from two aspects. The heterogeneity between policy agenda and public agenda is the first one. Most studies in China focus on it and find that the government can make policy formulation closer to public opinion by timely understanding public concerns and making policy adjustments [8,21]. Li found that the government’s grand policy narrative may not be conducive to public recognition of the policy and thus failed to enhance the public’s willingness to have children effectively [22]. Yang et al. extracted the content of citizen feedback on the bicycle-sharing policy based on text mining, compared it with the actual revision of the policy, and assessed the effectiveness of the government’s adoption of public opinion [23]. Huang et al. developed the research framework of public perception of innovation policies, analyzed the differences between public attention and policy concerns by taking the new energy vehicle policy as an example, and proposed corresponding policy suggestions [24]. The second aspect is the heterogeneity between media and public agendas. A few studies compared public concerns and media propaganda regarding policy information. Zhao et al. proposed a framework for public policy text analysis on social media and used machine learning to compare the policy information propaganda of online mainstream media with public opinion, finding a significant deviation between the media and the public [25]. In addition, studies on policy information diffusion using government social media mainly focus on policy diffusion strategies and effects. Jia et al. analyzed the current situation and main policy communication strategies on government Weibo, taking Shanghai’s garbage sorting policy as an example [26]. Ji et al. found that central and local governments’ garbage sorting policy advocacy through government WeChat presents new patterns different from traditional policy advocacy. The government overcame the previous top-down “command-and-control” model, with each focusing on strategy [27].

Existing studies showed an apparent heterogeneity in public policy information diffusion, and much attention is paid to the difference between the policy and public concerns. The research method gradually changes from traditional qualitative analysis of small sample data based on public opinion surveys and structured interviews to semantic mining based on social media data and applying data analysis methods such as machine learning. A few studies focused on the heterogeneity between the media and public agenda, and even fewer focused on the agenda of government social media. In addition, studies on policy information diffusion using government social media mainly focus on communication strategies and effects and mostly use qualitative case studies. However, exploring and comparing the policy interpretation of government social media with public concerns helps promote policy implementation, public participation, and government image building. Based on the above analysis, this study proposes the first research question:

**RQ1:** What issues are discussed on social media between government microblogs and the public around the specific public policy, and how does the level of attention to different issues differ?

### 2.2. Three Levels of Agenda-Setting Theory

The origin of agenda-setting theory is the “mimetic environment” mentioned by Walter Lippmann in “Public Opinion”, in which the public lives in the world presented by the media and is guided by the media agenda [28]. Maxwell McCombs and Donald Shaw conducted the chapel hill study in 1968. They found that the salience of issues on the public agenda was highly correlated with those on the media agenda, i.e., the media can determine what the public thinks [29]. That is the first level of agenda-setting theory, also known as object agenda-setting. For example, the frequent sharing of “data information” on social media by government microblogs may lead the public to believe it is a priority issue for public policy and should pay more attention to it. Later, in the 1995 Spanish elections, it was found that the emotional attributes of issues emphasized in media can be transferred to the public agenda, i.e., the media can also influence how the public thinks [30]. That is the second level of agenda-setting theory, also known as attribute agenda-setting. Traditional agenda-setting theory emphasizes the linear transfer of issues’ or attributes’ salience from the media to the public agenda. QAP correlation analysis is used to explore the correlation between the media and the public agenda. If there is a positive correlation between them, media agendas significantly influence public agendas [31].

Faced with the challenges of the new media environment, traditional agenda-setting theory, which tracks discrete objects or attributes that appear in news reports and public opinion polls, is insufficient to capture the current complex media environment. The emergence of the Internet transforms the information flow and access from the linear model to the network structure, bypassing the press as an intermediary and directly connecting different subjects [10]. In addition, the cognitive structure of human beings in the process of acquiring information and forming cognition is not linear but close to the network structure, in which different nodes are connected to form a cognitive map [11]. The news media is a critical factor influencing the public’s cognitive map, especially in public affairs. Therefore, NAS theory is proposed based on the characteristics of omnidirectional information flow in the Internet era and the theoretical foundation of cognitive networks. However, unlike traditional agenda-setting theory, NAS theory focuses on the association between issues or attributes rather than individual elements [32,33] and more accurately reflects the public’s cognitive structure. Guo et al. explored the role of news media in the public’s cognitive network. They found that the more frequently two elements in the news coverage are connected, the more likely the public perceives them as interrelated [34], i.e., the media also influences how the public relates different elements of the message [35]. That is the third level of agenda-setting, also known as NAS. Social network analysis is drawn extensively in the NAS model. The square co-occurrence matrix (i.e., a matrix with the same number of rows and columns) represents the agenda network. The entry in each cell in the matrix indicates the frequency of two related issues or attributes co-occurrence in the data. The higher the value, the stronger the correlation between them. Social network analysis and visualization illustrate how issues or attributes are related, in which nodes represent issues or attributes, and the edges between any two nodes represent the connection between them, with the higher the frequency of co-occurrence, the thicker the line [36]. Degree centrality replaces frequency as the primary measure of elemental salience [37]. Moreover, the statistical tools of network analysis can measure the similarity between different networks, thus exploring the extent to which the media transform the relational salience of objects or attributes to the public. Specifically, the QAP correlation test is used to analyze the similarity between the two matrices to measure the correlation between the media and public agenda networks.

Based on the above analysis, NAS theory has formed a relatively complete research system, including theoretical frameworks and research methods, drawing on the theoretical foundation of cognitive networks and social network analysis methods. Meanwhile, in the research area of international political communication [38], cross-cultural communication [39], and emergency public event information communication [40,41], studies verify the hypothesis of NAS theory. The results show that in the social media era, the public agenda and media agenda at the same time node have a co-temporal characteristic, i.e., the public agenda network and media agenda network are significantly and positively correlated. However, in the research area of public policy communication, the relationship between government microblog agendas and public agendas needs to be further explored. Therefore, based on the NAS theory, this study combines data mining, social network analysis, and visualization analysis to measure the phenomenon of agenda network heterogeneity between government microblogs and the public in multiple dimensions. Specifically, the second research question is proposed:

**RQ2:** What is the correlation between the GMANs and PANs, and what is their degree of heterogeneity in public policy communication?

### 2.3. Public Policy Communication and NAS

Social media is increasingly crucial in agenda setting, making large-scale communication possible. In the public policy communication of government, government microblogs enrich the channels of interaction between the government and the public, make it more convenient for the public to access information and participate in policy discussions, and improve the government’s responsiveness. The government microblog and public agendas influence each other through the online platform. Existing studies about agenda-setting focus more on political issues [42], especially the influence of media on voters’ voting [34]. Researches about public policy issues mainly focus on health care, especially public health issues in Asia, Africa, and Latin America, and the research horizon has gradually narrowed [37]. With the explosion of the COVID-19 epidemic, public health issues on social media have received much attention and become a critical point of public policy agenda-setting research in recent years.

Agenda-setting studies in crisis explore the influence of media agendas on public agendas and the dynamic guiding relationships from three levels of agenda-setting. Among them, studies on the NAS effect of media on the public mainly focus on the guiding relationship between influential news media and the public agenda. Han et al. found that they discuss a diversity of topics, and only a few issues are similar by analyzing the Twitter postings of the news media and citizens in the United Kingdom during COVID-19 [43]. Such studies focus on the first level of agenda-setting theory, i.e., whether news media coverage of COVID-19 influenced the public discussion of the issue. However, studies related to government social media mainly focus on the government’s public policy communication strategies. Li et al. explored what communication strategies were used by all levels of government (central, provincial, and municipal) in China to communicate with the public in response to the crisis. The results found that local governments mainly used guidance and investigation information strategies, while the central government mainly used support and advocacy information strategies. The central and local governments worked in concert and thus effectively responded to the crisis [44]. Wang et al. analyzed the posting content and frequency of the two most active government microblog accounts to explore how the Chinese government used social media accounts to communicate with the public during COVID-19. The study found that government microblog accounts posted frequently during the first outbreak of the epidemic and mainly posted virus-related information, China–US relations, and praise for medical personnel [9]. Such research focused on the government media’s public health policy coverage strategies, i.e., which attributes of policy information are emphasized in the media coverage. They did not investigate whether the media’s attribute emphasis influences the salience of attributes in the public agenda, focusing on the second level of agenda-setting theory. Moreover, Wang et al. investigated the effect of third-level dynamic agenda-setting between the news media and the public regarding the wildlife issue on social media through time series analysis. The findings suggest that the agenda-setting effect between the two is not unidirectional, and both interact with each other [13]. Only a few studies explored the guiding relationship between government social media agendas and public agendas in crisis. Iman et al. investigated the public health policy agenda-setting relationship between the World Health Organization and its different types of followers on Twitter. The novel findings of this research confirmed a “2-way” or “multiway” effect of agenda setting on social media due to the interactions between the content creators and audiences [14]. Dai et al. compared government-led agenda and public-led agenda setting during the COVID-19 pandemic in China to investigate whether or not the pandemic enhances the government’s role in agenda setting. It was found that the public agenda led the government agenda, and the government paid attention and responded to citizens’ emotions expressed through social media in crisis [15].

Existing studies show that the agenda-setting effect between the media and the public is not one-way, and both interact. Studies on government social media agenda setting mainly focused on the media’s coverage strategies on public health policy issues. Only a few studies focus on the agenda-setting effect and the guiding relationship between government social media and the public in a crisis. However, no consistent findings were obtained on their agenda-leading relationship. Therefore, it is urgent to investigate the role of government social media in agenda setting and clarify the agenda-leading relationship between them in public policy communication. Based on this, this study proposes the third research question:

**RQ3:** Do GMANs effectively lead PANs or vice versa in public policy communication?

### 2.4. Information Diffusion on Social Networks and NAS

In the era of intelligent media, the omnidirectional flow of information dramatically changes the communication mode between subjects, and the interaction between subjects forms the dynamic evolution of public opinion. Several crucial research problems exist in information diffusion on social networks based on different application scenarios [45]. For instance, identifying important nodes in a given network by defining and applying centrality measures, identifying bridge nodes in the network, guessing which non-adjacent nodes are likely to become adjacent by predicting links, etc.

Researchers from different fields have conducted extensive research on the above research problems. In the NAS of media communication, the concept of centrality is widely used, e.g., degree centrality, eigenvector centrality [46], etc., allowing researchers to examine which elements are at the center of news coverage retained by the audience. Compared with the previous two stages of agenda-setting theory, which use frequency as a measure, centrality provides a more comprehensive perspective to help researchers evaluate the status of different elements in the public cognitive system in a more macro context. In marketing advertising, Vikatos et al. proposed an approach based on a classification model to identify potentially influential bridge users by combining the network topology, linguistic traits, behavior, and multilayer features. This method achieves better performance in link prediction and benefits enterprise information dissemination, thus, brand and product promotion [47]. Moreover, Corradini et al. proposed the concept of k-bridge, i.e., users who connect k sub-networks of the same network or k networks in a multi-network scenario. Based on the anti-monotonic property of the k-bridge, a detecting algorithm for k-bridge nodes from social networks is proposed. The algorithm is applied to Yelp, Reddit, and other platforms to verify the universality of k-bridge features. k-bridges are also used to find the best marketing campaign targets and new products and services [48]. In online public opinion control, Qian et al. pointed out that bridging nodes, i.e., nodes with a small degree but connecting different groups within the network, sometimes play a decisive role in opinion evolution. Therefore, this study proposed an adaptive bridge control strategy for opinion evolution on social networks, which controls a special node called the bridge [49]. However, different studies define bridge nodes differently. Gao et al. proposed a community bridge-boosting prediction model and only focused on the bridge nodes connected to many communities. They ignored the bridge nodes with a low degree. The bridge nodes with the most connections are located in one community, while a small proportion of connections with other communities are also ignored [50]. In the global health crisis, a new bridging performance measurement was based on social connections to identify amplifiers in social media [51]. Furthermore, Bouyer et al. proposed a method to identify a group of influence-maximizing nodes by detecting overlapping communities, weighting the communities, and analyzing the emotional relationship of community nodes to identify influential people. It can be applied to helping companies commercialize, improving recommender systems, controlling rumor spread, and identifying immunization or quarantine targets to prevent epidemics in the population [52]. Regarding opinion leader identification, Bamakan et al. pointed out that it is an important task and summarized the corresponding identification methods [53]. Existing researchers effectively proposed approaches to identify central nodes in the real world [54] and mobile social networks [55]. The concept of top-k nodes is also proposed to identify the influential actors in a social network [56]. Recently, an exclusive graph neural network (GNN) model for opinion leader identification has been proposed, which utilizes the power of GNN to classify opinion leaders and their influence on online social networks [57]. Notably, a method is proposed to assess a post’s electronic Word of Mouth (eWoM) power, i.e., the post’s ability to be disseminated and known in social networks. Considering the traditional parameters that affect the post’s eWoM power, such as the number of comments, the number of retweets, etc., the time factor is also innovatively considered, thereby determining the main characteristics of successful posts. It plays a vital role in various applications, such as digital marketing, disseminating political and social opinions, and identifying the right influences [58].

Based on the above analysis, the existing studies define the importance of user nodes and the role of bridge nodes based on different application scenarios. The research methods are more suitable for large-scale real online social networks, with continuous optimization in efficiency and running time. However, traditional centrality measures, e.g., degree of centrality, are mainly used in existing NAS research to explore which issues are at the center of the agenda and more widely involved in connecting to other issues. This method overemphasizes the local characteristics of the network and ignores the global topology of the network. Therefore, we draw on the thought of bridges in the social network information diffusion and use FS entropy to comprehensively reflect the structural characteristics of network connection from the perspective of network flows. See Section 3.5.2 for more details. Therefore, combining the measure of issue importance derived from information entropy and traditional degree centrality enriches the research path of agenda-setting theory. Furthermore, by integrating the classical agenda-setting research methods, namely QAP correlation and regression analysis, we better explore the heterogeneous diffusion phenomenon between government microblogs and the public from multidimensions.

## 3. Research Methodology and Procedure

**Figure 1:**
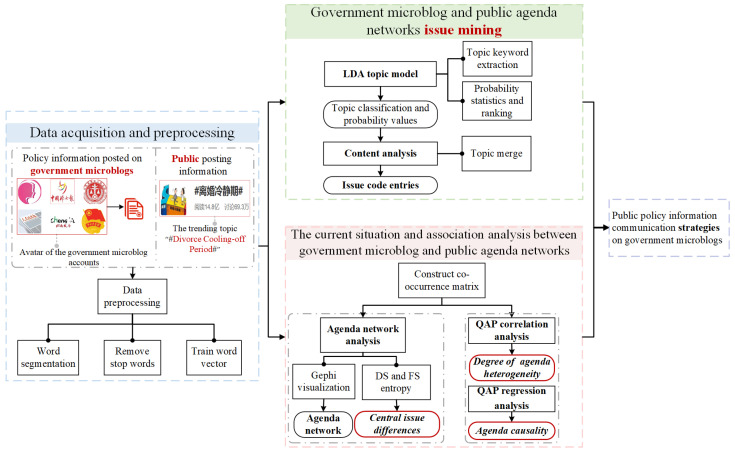
The research framework of the heterogeneous diffusion of public policy information on government microblogs.

We construct a research framework for the heterogeneous diffusion of public policy information on government microblogs, and the analysis process is shown in Figure 1. Firstly, the specific public policy information posted on the government microblog accounts and the public posting information under relevant trending topics on microblogs are collected and text preprocessed. Secondly, the LDA model is used to mine the obtained text data. Based on the output of the model, two coders independently code and merge the topics with similarity to obtain the discussed issues of government microblogs and the public on the specific policy. Further, the agenda co-occurrence matrix of government microblogs and the public is constructed, and their agenda network is visualized by Gephi software based on their respective matrices. The importance of the topics is measured by node importance analysis in DS and FS entropy. Then, the correlation between GMANs and PANs is examined by QAP correlation analysis to measure the degree of agenda heterogeneity between them. Meanwhile, QAP regression analysis investigates their causal relationship from a time-series perspective. Finally, the agenda network heterogeneity between government microblogs and the public is measured in three dimensions: difference in issue focus, degree of agenda heterogeneity, and agenda causality.

### 3.1. Case Selection

The case study selected for this research is China’s divorce cooling-off period policy. The Civil Code of the People’s Republic of China has been in effect since 1 January 2021. It provides that within thirty days from the date of receipting the divorce registration application from the marriage registration authority, any party who does not wish to divorce may withdraw this application [59]. The implementation of this policy received wide public attention and caused a great reaction in society. The related policy topics attracted public discussion and became trending on microblogs in March, April, and May. Disseminating policy information and responding to citizens on government microblogs have an essential impact on public opinion. Therefore, we select the divorce cooling-off period policy as a case study to validate the research framework for the heterogeneous diffusion of public policy information on government microblogs. Specifically, we analyze the heterogeneity between GMANs and PANs in public policy communication.

### 3.2. Data Acquisition and Preprocessing

We select government microblog posts and public posts about the divorce cooling-off period policy as the research object. Thirty-one government microblog accounts were chosen according to the top ten court microblogs, the top ten women’s federation microblog in China, and other lists in the government microblog influence ranking released by People’s Daily Online Public Opinion Data Center [60]. We searched for government microblog posts using “divorce cooling-off period”, “divorce”, and “cooling-off period” as keywords. Trending topic is the most critical type of interest page on the microblog, where the public can post microblogs and actively participate in discussions. Therefore, this study selected the trending topic “#Divorce Cooling-off Period#” hosted by People’s Daily to obtain public posts.

Since the implementation of the divorce cooling-off period policy on 1 January 2021, with the disclosure of divorce rate and other related data by government microblogs after the implementation of the policy and the exposure of domestic violence and cheating cases by the public, the associated topics of policy become trending on microblogs in March, April, and May and aroused heated public discussions. Therefore, we set the data analysis period as 1 January–31 May 2021, i.e., from the implementation of the policy to the time when the public discussion heated up and stabilized. Then, we used Octopus data collector software to crawl the above data. The obtained text data were manually processed to remove irrelevant and duplicate information. Eventually, we collected 610 posts from government microblogs and 2644 posts from the public. The text data inevitably contained invalid information, including emoticons, URL links, special symbols, etc. The corpus preprocessing module was used for basic cleaning and noise reduction. The precise mode of Jieba word splitting was used to slice the sentences. Meanwhile, we removed stop words and single words and combined high-frequency hyphenated words into one word for subsequent data analysis.

### 3.3. Topic Modeling

The LDA topic model is a versatile tool for identifying topics in social media text data, providing a probability distribution of different topics and their respective keywords. Notably, the model has no strict limitations on text length and has been widely used in topic mining in various domains [61,62]. LDA is a three-level Bayesian probability model, which belongs to a document topic generation model encompassing three granularities: document, topics, and keywords [63]. Its basic idea is that a document consists of different topics according to a specific probability distribution, and each topic consists of different keywords.

We use the LDA model to explore the discussion topics of the divorce cooling-off period policy on government microblogs and the public to obtain the object agenda. The model takes each posting from the public or government microblogs as a unit of analysis and outputs two probability distributions: document–topic and topic–keyword. The number of topics was determined by comparing the model’s perplexity under different topics. The final number of topics for the model was set to twelve according to the Elbow rule, i.e., a low degree of confusion and a small number of topics [64].

### 3.4. Co-Occurrence Matrix Analysis

According to the topic–keyword list output by the LDA topic model and the representative posts of each topic, two coders independently completed the content analysis of government microblogs and public posts. Meanwhile, the relevant keywords were merged into the same topic, and nine topic categories were summarized as the issue coding entries of the public policy agenda. The topics include policy information interpretation, premarital cooling-off period, litigious divorce, public opinion on policy, withdrawal of divorce registration application, data information, marriage and family counseling, domestic violence and cheating, and child parenting in the family, as shown in Table 1.

Based on topic modeling and manual coding, we transformed the document–topic matrix into a document–agenda matrix and constructed the agenda network based on the co-occurrence frequency of pairing issues in the same posts of government microblog and public. For instance, if a post on the government microblog contained “data information” and “withdrawal of divorce registration application”, there was a connection between the two topics. Five government microblogs and public agenda network matrices were constructed with the month as a time interval. Table 2 shows the public agenda network matrix for May 2021. Each row represents one topic, and the value of each cell in the matrix represents the number of times the pairing topics appear together in the post. The higher the value, the stronger the connection between the two issues.

### 3.5. Agenda Network Analysis

#### 3.5.1. Agenda Network Visualization

**Figure 2:**
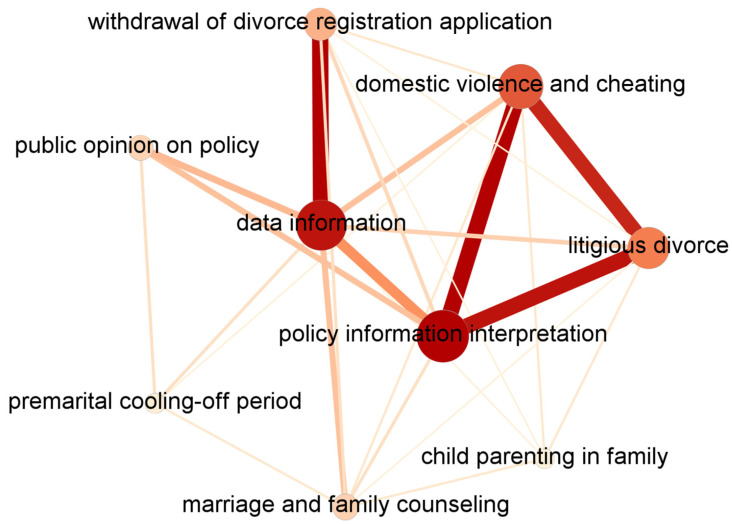
Government microblog agenda network, January to May 2021.

We imported the government microblog and the public agenda matrix into Gephi software to visualize the agenda network. Figure 2 shows the agenda network of government microblogs from January to May 2021. Each node represents an issue, and the connection between nodes represents the strength of the co-occurrence relationship between issues. The thicker lines between nodes, the higher the co-occurrence frequency of issues and the stronger the two issues are connected. The size of a node indicates its structural importance in the network. Section 3.5.2 describes the corresponding calculation method in detail. The larger the node, the higher the degree of centrality of the issue it represents and the more central it is in the network.

#### 3.5.2. Node Importance Analysis in DS and FS Entropy

Wu et al. use the relative degree of nodes to measure the node’s structure importance and propose the DS entropy to measure the network heterogeneity, formulated as follows [65].

(1) $$I_{k}=\frac{d_{k}}{\sum_{k=1}^{N}d_{k}}$$

(2) $$H=-\sum_{k=1}^{N}I_{k}\log I_{k}=-\sum_{k=1}^{N}\frac{d_{k}}{\sum_{k=1}^{N}d_{k}}\log\left(\frac{d_{k}}{\sum_{k=1}^{N}d_{k}}\right)$$

In Equations (1) and (2) above, $I_{k}$ is the node’s structure importance, H is the DS entropy, $d_{k}$ is the degree of node k, and N is the number of nodes in the network.

Cai et al. point out that it is more appropriate to use DS entropy to measure network heterogeneity [66]. Therefore, we use $I_{k}$ in DS entropy to measure the difference between government microblogs and public attention to different policy issues.

Freeman et al. also proposed a measure of betweenness centrality based on network flow, defined as flow betweenness. This measure permits using valued data on the strength of connections between different issues. Meanwhile, it can fully reflect the network’s global topology and evaluate a network node’s bridging capability. The flow betweenness is mathematically expressed as follows [67].

(3) $$C_{F}\left(k\right)=\frac{\sum_{i}^{N}\sum_{j}^{N}m_{k}(i,j)}{\sum_{i}^{N}\sum_{j}^{N}m(i,j)},where\;i\neq j\neq k$$

In Equation (3), the flow betweenness of node k is $C_{F}(k)$, N is the size of the network, m(i,j) is the maximum flow from node i to node j, $m_{k}\left(i,j\right)$ is the flow through node k in the maximum flow from node i to node j.

Furthermore, Cai et al. propose a maximum flow-based network structure entropy considering the network flow and flow betweenness—FS entropy. In FS entropy, the structural importance of node k is defined in terms of radial and medial measures. The medial measure is the absolute flow betweenness of node k, represented as follows [68,69].

(4) b′k=∑(i,j∈S(k))(Wi,jk−Wi,j*k)

In Equation (4), W is the maximum flow matrix between all node pairs in the network, Wk is the kth row and kth column removed from the matrix W, W*k represents the maximum flow matrix recalculated after removing node k from the original network. The radial measure ${d^{\prime }}_{k}$ is the degree of node k, which reflects the connection between a given node and other nodes in the network. Combining medial and radial measures, the structural importance of node k is defined as follows.

(5) $$I_{FS}\left(k\right)=\frac{I_{FS}^{\prime }\left(k\right)}{\sum_{n=1}^{N}I_{FS}^{\prime }\left(n\right)}=\frac{\alpha {b^{\prime }}_{k}+\beta {d^{\prime }}_{k}}{\sum_{n=1}^{N}\left(\alpha {b^{\prime }}_{n}+\beta {d^{\prime }}_{n}\right)},where\;\frac{\beta }{\alpha }=1$$

Specifically, the medial and radial measures were integrated to measure the structural importance of the issue in the agenda network. The medial measure reflects the dependence of the maximum network flow on the issue *k*, measures the number of paths through the issue, and evaluates the bridging ability of the issue. The radial measure reflects the connection between a given issue and other issues in the network. It assesses the group membership. The medial and radial measures complement each other to form the contribution of a given issue to the agenda network.

Based on the above analysis, we use $I_{k}$ in DS entropy and $I_{FS}\left(k\right)$ in FS entropy to measure the node k’s importance. Thus, we explore the differences in the attention that GMANs and PANs pay to each policy issue.

### 3.6. Correlation and Regression Analysis of Agenda Networks

QAP is a statistical method commonly used in NAS research. The correlation between two agenda networks was explored by comparing the values of cells in two matrices and calculating the Pearson correlation coefficient between the two matrices. When the two matrices significantly correlate, QAP regression analysis tests whether the independent variable predicted the dependent variable [70]. We used QAP correlation analysis to explore the correlation between government microblogs and public agenda co-occurrence matrix and measure the degree of heterogeneity between them based on the results of QAP correlation analysis.

QAP regression analysis has the same purpose as traditional regression analysis, i.e., to explore the causal relationship between relational variables. We further used QAP regression analysis to explore the leading relationship between GMANs and PANs. Specifically, we selected the earlier government microblog and later public agendas to explore how GMANs affect PANs. According to the occurrence of policy trending topics, we chose monthly as the period, and the whole time interval was divided into five time periods. Then, five correlation tests and four regression analyses were conducted on the data from the five time periods. From the time series perspective, the causal relationship between GMANs and PANs was judged by the time lag effect.

## 4. Results

### 4.1. GMANs and PANs Analysis

Figure 2 and Table 3 show the three core issues in the GMANs related to the divorce cooling-off period policy “policy information interpretation”, “data information”, and “domestic violence and cheating”. Meanwhile, all three had greater bridging power in GMANs, with closer connections to other issues. The line between “data information” and “withdrawal of divorce registration application” is thicker, indicating that government microblogs release the data information on the withdrawal of divorce registration application to emphasize the implementation effectiveness of the divorce cooling-off period policy. However, the “premarital cooling-off period” and “child parenting in the family” were weakly connected to other issues, and both were at the edge of the GMANs.

**Figure 3:**
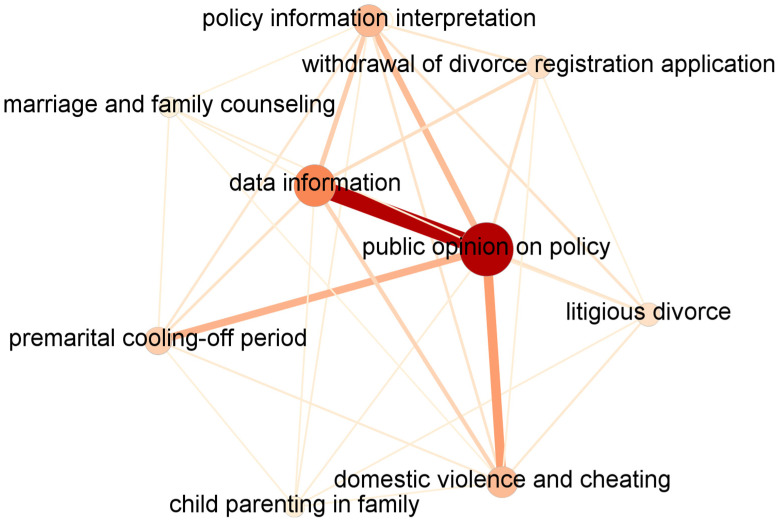
Public agenda network, January to May 2021.

Figure 3 and Table 4 show the public agenda network from January to May 2021, in which “public opinion on policy”, “data information”, “policy information interpretation”, and “domestic violence and cheating” are the four core issues. Among them, the first two issues had a greater bridging capability in PANs and stronger links with other issues. The links between “public opinion on policy” and “data information”, “premarital cooling-off period”, and “domestic violence cheating” are thicker, indicating that these three groups of issues are closely linked together. Moreover, the importance of the “premarital cooling-off period” issue increased in PANs, reflecting the public’s call for implementing a premarital cooling-off period policy rather than the divorce cooling-off period policy.

### 4.2. Correlation Analysis of GMANs and PANs

QAP correlation analysis was used to examine the correlation between the GMANs and the PANs within the same interval to answer research question 2. As shown in Table 5, at the beginning of policy implementation in January and February, there was a significant correlation between the GMANs and the PANs, and the degree of heterogeneity between them was relatively small. However, with trending topics emerging in March, April, and May, GMANs and PANs did not significantly correlate and showed considerable differences, with greater heterogeneity between them.

Specifically, after the divorce cooling-off period policy is implemented for some time (In March, April, and May), the government microblogs release positive information about the implementation effect of the policy, such as the decrease in the divorce rate and the number of withdrawn divorce registration applications. However, the public debate the negative impact of policy implementation, with trending topics such as “#31 Provincial Marriage and Divorce Big Data”, “Jin Jiang Yue Shi Shui”, and “Divorce Number Decreased by 70% in the First Quarter of China”, respectively. The public expresses strong dissatisfaction with the problems such as domestic violence and cheating, failed divorce due to withdrawal of divorce registration application by only one party, fear of marriage and childbirth, and long litigated divorce cycle after the implementation of the policy. They also question the data released by government microblogs, such as the number of withdrawn divorce registration applications and the declining divorce rate. Meanwhile, the public calls for freedom of marriage and establishing a premarital cooling-off period.

### 4.3. The Impact of GMANs on PANs

For research question 3, we first explore the influence of GMANs on PANs. The research results are shown in Table 6. The GMANs have some effect on PANs. Specifically, we conduct four QAP regression analyses between the GMANs and the PANs, among which two QAP regression analyses are significant. The first is that government microblogs agenda in January significantly impacted the public agenda in February, with a regression coefficient of 0.538 (*R*^2^ = 0.290, *p* = 0.018). The second is that government microblogs agenda in February greatly influenced the public agenda in March, with a regression coefficient of 0.623 (*R*^2^ = 0.388, *p* = 0.001). However, after a period of policy implementation (after March), with government microblogs’ disclosure of data information and the exposure of cases such as domestic violence during the divorce cooling-off period, GMANs no longer significantly impact the PANs.

Specifically, at the end of March, government microblogs disclosed data such as the abandonment of divorce registration during the divorce cooling-off period in various provinces, which caused heated public discussion and formed a trending topic, namely “#31 Provincial Marriage and Divorce Big Data”. The public doubted the authenticity of the number of withdrawn divorce registration applications. They also expressed strong dissatisfaction with the policy that any party who does not want to divorce can withdraw the divorce registration application from the marriage registration authority. In April, the cheating and domestic violence during the divorce cooling-off period aroused strong public dissatisfaction with the policy implementation, resulting in the trending topic “#Jin Jiang Yue Shi Shui”. In May, government microblogs revealed the number of divorces in the first quarter of China, which led to public debate and the trending topic “Divorce Number Decreasing by 70% in the First Quarter of China”. The majority of the public expressed strong dissatisfaction with the purpose of the divorce cooling-off period policy to reduce the divorce rate. They believed that the decline in the divorce rate is related to the death of domestic violence, the failure of divorce caused by the withdrawal of divorce registration application by only one party, and the lengthy divorce litigation. Meanwhile, they expressed sentiments such as fear of marriage and called for a premarital cooling-off period and freedom of marriage. The relevant public microblog posting is as follows:

木栖乐荟 (Sina microblog name): “#Divorce Cooling-off Period # right, so that more people die, more fear of marriage, and no more people get married, how good”.

In addition, the results in Table 7 show that the public agenda network significantly affects its agenda network in the next time interval during public policy communication. All four QAP regression analysis results were significant at the 0.001 level. Moreover, the regression coefficients were large, as shown in the first row of Table 7.

### 4.4. The Impact of the PANs on GMANs

For research question 3, the PANs’ impact on GMANs was secondly explored. The research results are shown in Table 8, where PANs had no significant influence on GMANs. Specifically, we conducted four QAP regression analyses between the PANs and the GMANs. Among them, the *p*-values of all four regression analysis results were greater than 0.05, i.e., none were significant, as shown in the first row of Table 8. Therefore, in the agenda of the divorce cooling-off period policy on the Sina microblog platform, PANs failed to affect GMANs, i.e., reverse agenda setting was ineffective. This finding suggests that government microblogs fail to promptly and effectively respond to the public’s demands and adjust the policy communication content on time, resulting in a significant deviation from the public.

In addition, the last two rows in Table 8 show that GMANs have a small impact on the agenda network in the next time interval during public policy communication. Specifically, three of the four QAP regression analysis results were significant at the 0.05 level, as shown in the second row of Table 8. For instance, the government microblog agenda in February significantly impacted its agenda in March, with a regression coefficient of 0.373 (*R*^2^ = 0.139, *p* = 0.039).

## 5. Discussion

Based on the NAS theory, we first explored what object agendas are presented by government microblogs and citizens in public policy information diffusion through topic mining and content analysis. Then, we used the importance of nodes in DS and FS entropy to measure their attention to different issues. Finally, the QAP correlation and regression analysis investigated the agenda network heterogeneity between government microblogs and the public. The study indicates that at the beginning of the policy implementation in January and February, there was a significant correlation between GMANs and PANs, with less heterogeneity between them. Moreover, GMANs led the PANs. However, with the emergence of trending topics in March, April, and May, there was no significant correlation between GMANs and PANs, with more significant heterogeneity between them. In addition, the GMANs do not effectively influence the PANs. Specifically, we find that government microblogs consciously focus on issues such as “policy information interpretation” and “data information” as the point of agenda setting. This finding shows that the government microblogs focus on interpreting and promoting the policy related to the divorce cooling-off period in the Civil Code and popularizing the policy’s relevant provisions and legal procedures. After implementing the policy, the government microblogs also disclose data on the number of withdrawn divorce registration applications and the divorce rate. The core issues of the public agenda are “public opinion on policy”, “data information”, and “domestic violence and cheating”. The public doubts the authenticity of the data information disclosed by the government, such as the number of withdrawn divorce applications, divorce rate, etc. They focus on the application of the policy and concern about domestic violence during the divorce cooling-off period and express strong dissatisfaction with the implementation of the policy. However, there is high public support for premarital cooling-off periods. Therefore, there is a significant difference in the agenda-setting of the divorce cooling-off period policy between government microblogs and public concerns. Furthermore, we find that the trending topics and public opinion on the policy do not significantly affect the government microblog agenda. This conclusion shows that in public policy information diffusion, government microblogs fail to listen to public opinion promptly, capture public attention, and form effective communication with the public, which affects the government microblogs to adjust communication strategies timely and effectively.

This study provides specific references for public policy diffusion on government microblogs in research methods, theoretical applications, and practical significance.

Firstly, at the level of research methodology. Based on NAS theory, we combined a machine learning model with manual coding, i.e., topic modeling and content analysis, to mine the policy discussion topics between government microblogs and the public. The $I_{k}$ of DS entropy and $I_{FS}(k)$ of FS entropy measure the differences between government microblogs and public attention to different issues. The QAP correlation analysis measures the degree of heterogeneity between the two agenda networks. Further, QAP regression analysis explores the causal relationship between the two agenda networks. Overall, we measure the heterogeneity between government microblogs and public agenda from three dimensions: differences in issue attention, degree of agenda heterogeneity, and agenda causality.

Secondly, at the level of theoretical applications, this study extends the NAS theory to the research field of public policy information diffusion on government microblogs. We investigate the heterogeneity between the interpretation of policy on government microblogs and the public’s attention to policy, which somewhat broadens the interpretation scope of this theory. First, the empirical study constructs a framework to clarify the heterogeneous diffusion phenomenon between government microblogs and the public in three dimensions: differences in issue attention, degree of agenda heterogeneity, and agenda causality. Second, we extend the NAS theory from a single temporal cross-section to a time sequence perspective and analyze the issue leading to the relationship between GAMNs and PANs. Third, previous studies mainly focus on government social media coverage strategies on public health policy issues. We regard government microblogs as the main body of agenda-setting and explore the agenda-setting effect and the guiding relationship between government microblogs and the public in public policy communication.

Finally, this study has some guiding significance for the future practice of public policy information diffusion on government microblogs. The following strategies for public policy information diffusion on government microblogs are proposed based on the above research findings.

1. Government microblogs focus on “policy information interpretation”, while the public’s understanding of policy information is biased. This phenomenon may be due to the rigid form of policy propaganda and lack of policy information interaction, which causes the gap between policy interpretation of government microblogs and public policy understanding. Thus, it affects the public’s cognition and evaluation of public policies and may generate negative emotions due to bias in understanding or conflicts of interest. Given the above problems, the following communication strategies can be adopted by government microblogs. First, policy information diffusion should be media-oriented, maximizing the government’s media communication resources. In addition to traditional texts and pictures, use videos, films, motion graphics, big data, virtual reality, games, hyperlinks, and other media forms as needed. Second, the government takes the initiative to set issues and make them trending topics. Furthermore, the government invites the public to participate in discussions and accepts “reverse invitations” to directly join the public-led online discussion, promoting the interaction of policy communication.
2. Government microblogs fail to guide PANs effectively. Although both government microblogs and the public are concerned with data disclosure, government microblogs focus more on positive data information, such as the decline in the divorce rate and the number of withdrawn divorce applications after the implementation of the policy. They aim to clarify the effectiveness of the divorce cooling-off period policy by releasing positive information to improve the public’s recognition of the policy. However, the public is still skeptical about implementing the policy from the perspective of personal interests and believes that the implementation of the policy may decrease the marriage rate. Therefore, the government should reasonably guide the public to correctly perceive the policy through government microblogs and other media, effectively propaganda the significance of the policy implementation, and timely answer the public’s doubts about the policy. Specifically, the government should first set up a multi-framework to interpret the policy from the perspective of realizing the people’s livelihood interests, the residents’ quality of daily life improvement, etc., rather than a grand narrative. Second, the government should adopt popular discourse to realize policy intention. For instance, using novel headlines, Internet buzzwords, and emoticons adds vitality to policy discourse. The distance between the policy disseminator and the policy audience can be dissolved through the novel and friendly discourse. It is conducive to the public’s understanding and acceptance of the policy and helps ease the social emotions arising from differences in understanding or conflicts of interest.
3. The “public opinion on policy” is the public’s greatest concern, but government microblog attention is relatively low. Moreover, government microblogs fail to listen to public opinions and capture public concerns in time, thus affecting the timely and effective adjustment of their policy information diffusion strategies. The keywords “oppose, compulsory, freedom of marriage” reflect the negative attitude of a part of the public towards implementing the divorce cooling-off period policy and their opposition to the policy. At the same time, implementing the policy changes the public perception of marriage. The keywords “marriage, caution, no marriage“ reflect that the public is more cautious about marriage due to the restrictions on divorce imposed by the policy, which may decrease the marriage rate in China. It is worth noting that there is a strong voice supporting the implementation of the premarital cooling-off period policy. The policy advocates premarital medical examinations and safeguards spouses’ right to premarital information. Therefore, the government should listen and respond promptly to the public’s voice and consider gradually putting the “premarital cooling-off period” policy on the policy agenda. It will fundamentally help avoid impulsive divorce and reduce the divorce rate in China, thereby protecting marriage and family relations and maintaining social harmony and stability.

## 6. Conclusions

This paper constructs a research framework for the heterogeneous diffusion of public policy information on government microblogs. The heterogeneous diffusion phenomenon between government microblogs and the public is measured from three dimensions, i.e., differences in issue attention, degree of agenda heterogeneity, and agenda causality. The DS and FS entropy, QAP correlation test, and QAP regression analysis from the above three dimensions comprehensively explore the heterogeneous diffusion of GMANs and PANs. The results show that GMANs have some influence on PANs at the beginning of public policy implementation, and the degree of heterogeneity between them is small. However, with the government’s disclosure of information on the positive effect of public policy implementation, GMANs do not effectively affect the PANs, and the degree of heterogeneity between the two is relatively large. Furthermore, PANs do not significantly affect GMANs.

There are still some limitations in this study. On the one hand, we only focus on some active government microblogs posting information, and the data set needs to be expanded. Therefore, future research can continue to expand the scope of government microblogs and thus more fully reflect the policy information diffusion characteristics of government microblogs. On the other hand, future research can consider extending sentiment analysis to the analytical framework to explore the dynamic evolution of emotional agenda attributes among different subjects. Furthermore, a post’s eWoM power assessment [58] may be used in future work better to explore the communication of the government’s public policy opinions.

## Figures and Tables

**Table 1:** Topics in the government microblogs and public agenda.

Topics	Keywords	Government Microblog Posts (%)	Public Posts (%)
policy information interpretation	civil code, terms, content, legal literacy class, consent	21.5	7.4
premarital cooling-off period	marriage, caution, marriage cooling-off period, suggest, no marriage	1.5	6.4
litigious divorce	court, lawsuit, mediate, divorce, dispute	11.5	2.2
public opinion on policy	vote, topics, oppose, compulsory, freedom of marriage	8.5	53.2
withdrawal of divorce registration application	couples, divorce cooling-off period, withdrawal, marriage registration	13.8	2.2
data information	divorce rate, marriage rate, decrease, data, inaccurate	28.5	16.3
marriage and family counseling	Premarital education, counseling courses, marriage, family	3.1	1.4
domestic violence and cheating	domestic violence, situations, women, protection, cheating	10.8	9.2
child parenting in the family	divorce, minor children, parenting, children, marriage, breakdown	0.8	1.7

**Table 2:** Public agenda network matrix for May 2021.

	A	B	C	D	E	F	G	H	I
**A**		2	8	15	2	16	0	2	0
**B**	2		0	40	0	13	0	5	1
**C**	8	0		7	0	5	0	3	1
**D**	15	40	7		1	142	1	46	2
**E**	2	0	0	1		2	0	0	0
**F**	16	13	5	142	2		0	24	0
**G**	0	0	0	1	0	0		1	0
**H**	2	5	3	46	0	24	1		1
**I**	0	1	1	2	0	0	0	1	

NOTE: A = policy information interpretation; B = premarital cooling-off period; C = litigious divorce; D = public opinion on policy; E = withdrawal of divorce registration application; F = data information; G = marriage and family counseling; H = domestic violence and cheating; I = child parenting in the family.

**Table 3:** The importance of each issue in GMANs.

Issues in GMANs	The Importance of Each Issue in DS and FS Entropy	
	DS Entropy	FS Entropy
A	0.213	0.213
B	0.026	0.038
C	0.151	0.111
D	0.056	0.058
E	0.098	0.077
F	0.203	0.243
G	0.060	0.082
H	0.169	0.141
I	0.024	0.038

NOTE: A = policy information interpretation; B = premarital cooling-off period; C = litigious divorce; D = public opinion on policy; E = withdrawal of divorce registration application; F = data information; G = marriage and family counseling; H = domestic violence and cheating; I = child par-enting in the family.

**Table 4:** The importance of each issue in PANs.

Issues in PANs	The Importance of Each Issue in DS and FS Entropy	
	DS Entropy	FS Entropy
A	0.127	0.138
B	0.081	0.062
C	0.039	0.047
D	0.350	0.384
E	0.039	0.037
F	0.228	0.205
G	0.005	0.006
H	0.121	0.103
I	0.011	0.018

NOTE: A = policy information interpretation; B = premarital cooling-off period; C = litigious divorce; D = public opinion on policy; E = withdrawal of divorce registration application; F = data information; G = marriage and family counseling; H = domestic violence and cheating; I = child par-enting in the family.

**Table 5:** QAP correlation between GMANs and PANs.

Time Interval	QAP Correlation (Pearson’s r)
January	0.57 *
February	0.50 **
March	0.15
April	0.06
May	−0.03

NOTE: * *p* < 0.05, ** *p* < 0.01.

**Table 6:** QAP regression analysis between GMANs and PANs, the dependent variable is PANs.

	PANs				
	January	February	March	April	May
GMANs _t−1_		0.538 *	0.623 ***	−0.074	−0.058
Adjusted R-squared		29.0%	38.8%	0.5%	0.3%

NOTE: * p < 0.05, *** p < 0.001; “GMANs _t−1_” refers to the government microblogs agenda network in the previous time interval.

**Table 7:** QAP regression analysis between the PANs and its agenda network in the next time interval.

	PANs				
	January	February	March	April	May
PANs _t−1_		0.856 ***	0.783 ***	0.872 ***	0.855 ***
Adjusted R-squared		73.3%	61.4%	76.0%	73.0%

NOTE: *** p < 0.001; “PANs _t−1_”, public agenda network in the last time interval.

**Table 8:** QAP regression analysis between GMANs and PANs, the dependent variable is GMANs.

	GMANs				
	January	February	March	April	May
PANs _t−1_		0.24	−0.04	0.33	−0.03
GMANs _t−1_		0.263	0.373 *	0.505 *	0.411 *
Adjusted R-squared		6.9%	13.9%	25.5%	19.1%

NOTE: * p < 0.05; “PANs _t−1_”, public agenda network in the last time interval; “GMANs _t−1_”, government microblog agenda network in the last time interval.

## Data Availability

Not applicable.
